# Supplementary material for: Sources of PCR-induced distortions in high-throughput sequencing data sets
Source: Nucleic Acids Res. 2015 Jul 17;43(21):e143. doi: 10.1093/nar/gkv717 (PMC4666380; doi:10.1093/nar/gkv717)
Supplement: SUPPLEMENTARY DATA [file supp_43_21_e143__index.html]

Sources of PCR-induced distortions in high-throughput sequencing data sets — SUPPLEMENTARY DATA 

# Sources of PCR-induced distortions in high-throughput sequencing data sets

## SUPPLEMENTARY DATA

- SUPPLEMENTARY DATA
- SUPPLEMENTARY DATA
