# Supplementary material for: Sources of PCR-induced distortions in high-throughput sequencing data sets
Source: Nucleic Acids Res. 2015 Jul 17;43(21):e143. doi: 10.1093/nar/gkv717 (PMC4666380; doi:10.1093/nar/gkv717)
Supplement: SUPPLEMENTARY DATA [file supp_gkv717_nar-01434-met-g-2015-File009.pdf]

Supplemental figures

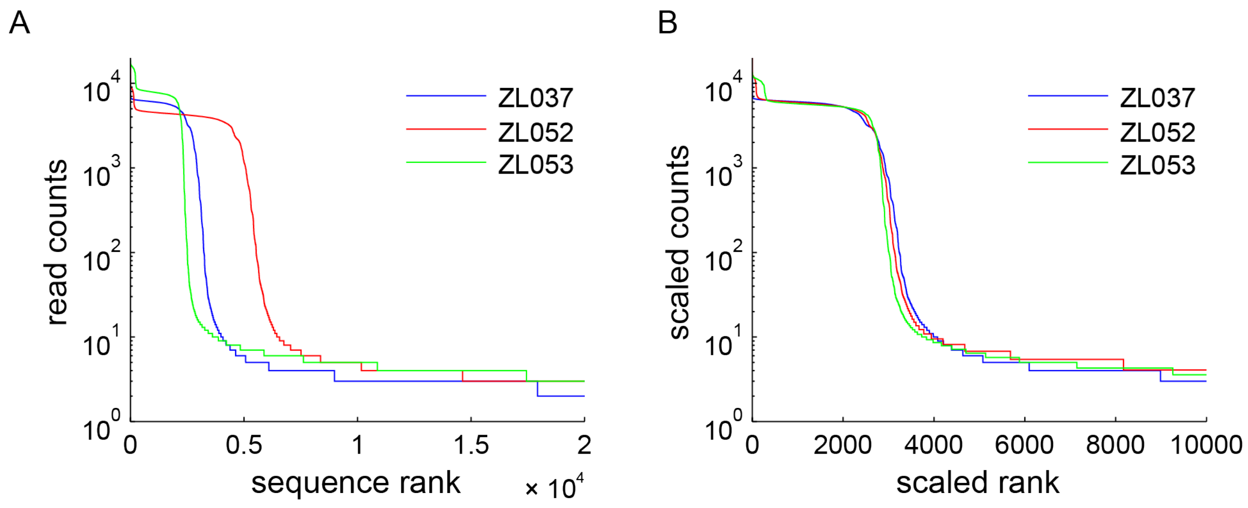

Figure S1. Sequence rank plot of replicate datasets ZL037, ZL052 and high GC dataset ZL053 before (A) and after (B) linear scaling in the x and y to compensate for different input amounts and sequencing depth. Scale factors for the x and y dimensions can be found in Table 3.
